# A Survey on Adaptive Data Rate Optimization in LoRaWAN: Recent Solutions and Major Challenges

**DOI:** 10.3390/s20185044

**Published:** 2020-09-05

**Authors:** Rachel Kufakunesu, Gerhard P. Hancke, Adnan M. Abu-Mahfouz

**Affiliations:** 1 Department of Electrical, Electronic and Computer Engineering, University of Pretoria, Pretoria 0002, South Africa; u10399764@tuks.co.za (R.K.); a.abumahfouz@ieee.org (A.M.A.-M.); 2 Nanjing University of Posts and Telecommunications, Nanjing 210023, China; 3 Council for Scientific and Industrial Research, Pretoria 0184, South Africa

**Keywords:** adaptive data rate, algorithm, Internet of things, LoRa, LoRaWAN, LPWAN

## Abstract

Long-Range Wide Area Network (LoRaWAN) is a fast-growing communication system for Low Power Wide Area Networks (LPWAN) in the Internet of Things (IoTs) deployments. LoRaWAN is built to optimize LPWANs for battery lifetime, capacity, range, and cost. LoRaWAN employs an Adaptive Data Rate (ADR) scheme that dynamically optimizes data rate, airtime, and energy consumption. The major challenge in LoRaWAN is that the LoRa specification does not state how the network server must command end nodes pertaining rate adaptation. As a result, numerous ADR schemes have been proposed to cater for the many applications of IoT technology, the quality of service requirements, different metrics, and radio frequency (RF) conditions. This offers a challenge for the reliability and suitability of these schemes. This paper presents a comprehensive review of the research on ADR algorithms for LoRaWAN technology. First, we provide an overview of LoRaWAN network performance that has been explored and documented in the literature and then focus on recent solutions for ADR as an optimization approach to improve throughput, energy efficiency and scalability. We then distinguish the approaches used, highlight their strengths and drawbacks, and provide a comparison of these approaches. Finally, we identify some research gaps and future directions.

## 1. Introduction

Increasingly, organizations in many industries are employing the Internet of Things (IoT) to function more efficiently, to understand their customers better, to provide improved customer service, enhance decision-making and boost the value of the business. This has resulted in a considerable upsurge in the number of devices connected to the internet. A “thing” in the IoT, can be a patient implanted with a heart monitor, smart home that automatically regulate heating and lighting, a farm animal with a biochip transponder, a smart city or a smart factory monitoring industrial machines searching for glitches, then automatically regulates to avoid breakdowns [1,2,3,4]. These devices need to be capable of getting an Internet Protocol (IP) address assigned to them and possess the ability to transfer data over a network. Low Power Wide Area Networks (LPWAN) is a novel kind of wireless communication, attributed to low power, low bit rate and long-range communication technologies in the unlicensed industrial, scientific and medical (ISM) frequency bands, configured in a star topology network. This makes it suitable for many IoT applications that simply require transmission of small data packets over long distances. LPWAN allows long-range transmission up to ten to forty kilometres in rural environments and one to five kilometres in urban environments [5]. LPWAN is extremely scalable and energy efficient [6]. In the LPWAN space there are contending technologies, for instance Sigfox, NB-IoT and Long-Range Wide Area Networks (LoRaWAN). A comparison of these LPWAN technologies is found in [7]. In this review paper the focus is on LoRaWAN, which exhibits the attributes of straightforward deployment, low power consumption, and long-distance coverage. LoRaWAN is an open LPWAN standard that uses unlicensed spectrum. LoRaWAN is a key technology for the IoTs, suitable for a variety of fixed and mobile IoT applications.

An essential LoRaWAN feature is the Adaptive Data Rate (ADR) scheme. The objective of this scheme is to minimize energy consumption and maximize throughput by adjusting the data rate dependent on the link budget for every end node in a LoRaWAN. ADR controls the transmission parameters, namely Bandwidth (BW), Spreading Factor (SF), Transmission Power (TP) and Coding Rate (CR). Optimizing the ADR increases the network capacity since data packets transmitted with different SFs are orthogonal and therefore can be received concurrently, thus reducing airtime. ADR is a mechanism that controls the uplink (UL) transmission parameters of LoRa devices depending on the link budget. The ADR functionality needs to be enabled at the end node for it to be employed. In the LoRa implementations found in the literature, ADR schemes are implemented and evaluated using different approaches such as mathematical models, simulations, and testbeds. The major challenge with the ADR algorithm is that the LoRa specification does not state the way the Network Server (NS) should instruct end nodes regarding rate adaptation. This has resulted in a gap in terms of ADR implementation as vendors keep their implementations private, consequently, many different ADR schemes have been proposed. The surge in IoT deployments results in different quality of service (QoS) requirements, metrics, and implementation approaches, which provide a challenge in the reliability and suitability of the ADR schemes. The main contributions of this paper are as follows:

- An overview of the LoRaWAN system and its parameters are thoroughly reviewed
- An investigation of the existing ADR schemes that have been proposed in the public domain and an evaluation of how these approaches address the challenges of ADR.
- A discussion of the strengths and drawbacks of the proposed schemes.
- Finally, the identification of research gaps and potential future direction.

The remainder of the paper is organized as follows: Section two presents a technology overview of LoRaWAN, describing the LoRa physical layer and the LoRaWAN medium access control (MAC) layer, the transmission parameters and how they apply to LoRaWAN network performance. Section three provides a review of the adaptive data rate algorithms. Section four presents a comparison and discussion of the ADR schemes. Section five explores research gaps and future direction. Section six concludes this paper. Appendix A provides Table A1 which lists the acronyms that are used in this paper.

## 2. Technology Overview

LoRaWAN protocol was first released in 2015 by the LoRa Alliance. The LoRaWAN functions in an unlicensed sub-gigahertz ISM band (863–870 MHz band in Europe and 902–928 MHz in USA). It uses the 125 KHz, 250 KHz and 500 KHz bandwidth and transmits payloads of up to 250 Bytes over 5–15 km. The system consumes low power and can last up to five to ten years in battery life [8,9]. LoRa, represents “Long-Range”, a long-range wireless communications technology, endorsed by the LoRa Alliance. The main objective of this technology is to be functional in long lasting battery-operated end nodes, where energy efficiency is utmost priority. LoRa consists of two distinct layers, namely a physical layer that uses the Chirp Spread Spectrum (CSS) radio modulation system and a MAC layer protocol known as LoRaWAN. However, the LoRa communication system furthermore denotes a particular access network architecture [10]. LoRaWAN is constructed to optimize deployment cost, capacity, range, and battery lifetime in LPWANs. LoRa, the physical layer or modulation employed to generate the long-range wireless connection, provides multiple transmission parameters. These parameters are BW, SF, TP and CR. Varying these parameters affects network performance.

**Figure 1:**
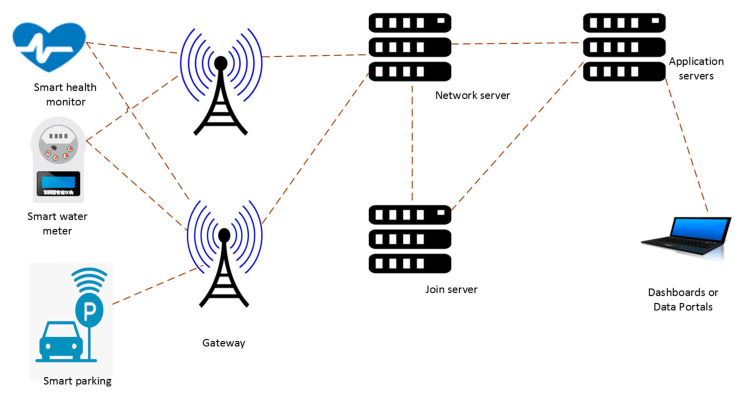
Long-Range Wide Area Networks (LoRaWAN) architecture [10].

The LoRa network comprises five main components: the end nodes also known as end devices, the gateway, the NS, the Join Server (JS) and application servers connected in a star topology architecture. The LoRa end node contains a wireless transceiver and sensor nodes that transmit data to multiple gateways in its vicinity using LoRa radio frequency (RF) modulation. Gateways are powered by the mains and have internet connectivity. They consist of a radio component with a transmitter and a microprocessor for data processing. Every gateway in the network sends the received data packet to the cloud-based NS which successively directs the packet to the appropriate application server. If a network has multiple gateways, it is possible for all the gateways to receive data from the same end node. Gateways can concurrently listen to several frequencies in every SF. Figure 1 shows the LoRaWAN architecture.

When an end node forwards a data packet to the gateway, it is known as an UL and when the gateway forwards a data packet to the end node, it is called a Downlink (DL). End nodes broadcast their data packets to every gateway in the vicinity and the gateways transmit the data to the NS. The NS sends the data package to the correct application server where the end-user can process the data. The NS receives the response from the application server and establishes which gateway will send the message back to the end nodes. Two types of messages can be transmitted at any given time in any LoRaWAN operation. These are unconfirmed messages, where the end nodes do not request a response from the NS, and confirmed messages which request a response. For the end nodes to be deemed active entities, they are required to first connect to the network and be allocated a series of parameters that are essential for operation in a LoRaWAN through the JS. The LoRaWAN network contains two major security elements, the join procedure and message authentication. This guarantees that only authentic and certified end nodes are joined to authentic and bona fide networks. The implementation of end-to-end encryption for application payloads that are exchanged between the end nodes and the application servers is also provided by LoRaWAN as a security measure. 

LoRaWAN is among a small number of IoT systems that implement end-to-end encryption. The JS is responsible for secure device activation and key storage and management. It indicates to the NS which application server should be connected to the end nodes and executes the encryption key derivations for the network and application session. The network session key of the end node is communicated to the NS, and the application session key to the corresponding application server. LoRaWAN permits two kinds of end device activation, namely Over-the-Air Activation (OTAA) and Activation by Personalization (ABP). The OTAA process permits end nodes to validate and secure access to the network with security credentials. ABP is a simpler and less secure process which skips the join procedure. The LoRaWAN protocol does not support direct communication between end nodes [10]. Security is a huge challenge in IoT networks. The authors in [11] provided an extensive security risk analysis of the LoRaWAN v1.1 protocol and discussed numerous countermeasures to the security risks outlined. They presented a “threat” catalogue, as well as propositions and analyses regarding the magnitude, effect, and possibility of each threat. Other suggestions of improving the network security have been suggested in [12,13,14].

### 2.1. LoRa

LoRa is the physical (PHY) layer of the LoRa technology which utilizes Chirp Spread Spectrum modulation and Forward Error Correction (FEC) that trades-off data rate for sensitivity, within a fixed BW. This combination enables long transmission ranges with low power characteristics as the chirp signals use the available bandwidth immediately. This characteristic makes the chirp signals robust from interference and noise. Each LoRa packet consists of a preamble of 10 chirps and six synchronization chirps followed by the data. Multiple data bits (chips) can be modulated by one chirp. The SF parameter determines the number of bits that are modulated. For instance, SF7 means a chirp can be encoded with seven bits. Transmitting a data packet with a higher SF implies additional bits are encoded into the chirp, resulting in additional Time on Air (ToA) and a reduced data rate, although it enhances robustness to noise. Three other parameters are involved in LoRa modulation that is BW, TP, and CR. Three bandwidth settings are available namely 125 KHz, 250 KHz and 500 KHz. The available code rate settings are 4/5, 4/6, 4/7 or 4/8 [15]. These parameters together are used to calculate the data rate, also known as the LoRa modulation bit rate $R_{b}$. Equation (1) expresses the relationship between data transmission rate and CR, BW, and SF [16].

(1) $$R_{b\;}=SF*\frac{BW}{2^{SF\;}}*CR,$$

where: 

SF = Spreading Factor

BW = Modulation Bandwidth

CR = Code Rate.

Tuning the parameters above results in a number of the end-to-end transmission characteristics such as data rate, error correction capacity and communication range to be variable [17]. Although a combination of SF, BW and CR are possible theoretically, practically, SF and BW are combined to form the data rate, depending on the LoRaWAN regional parameters specification [9]. The specification document specifies the different regulatory requirements of LoRaWAN depending on the location of the deployment. In North America, for instance, there are 64, 125 kHz LoRa UL channels that have been specified, centered on a 200 kHz raster. Eight 500 kHz UL channels as well as eight, 500 kHz DL channels have also been specified. The gateways have the capacity of up to 64, 125 kHz UL channels and eight 500 kHz UL and DL channels. In Europe, LoRaWAN defined ten channels, eight of which have multiple data rates from 250 bps to 5.5 kbps. The specification also has one high data rate LoRa channel at 11 kbps, and one frequency shift keying (FSK) channel at 50 kbps. The European Telecommunications Standards Institute (ETSI) allows maximum output power in Europe of +14 dBM, except the G3 band which allows +27 dBm. Duty cycle restrictions exist under ETSI but there are no limitations on maximum transmission or ToA. As an example, the regional settings EU863-870 used in Europe and US902-928 used in North America have the data rates ranging from SF7BW125 to SF12BW125 and SF7BW125 to SF10BW125, respectively. The ADR adjusts the data rate depending on the available link budget. The larger the SF applied, the further the signal travels and the less interference present at the receiver. With the bandwidth fixed at BW125 as given in the example, lowering the SF increases the data rate, thereby minimizing the ToA for data packets making the gateway more sensitive to noise. The end nodes that are nearest to a gateway transmit using the lowest SF thereby prolonging the battery life because of the effect of ToA. More distant sensors transmit at a higher SF but the data rate will be low. A compromise is made between battery power and range considering that a higher SF allows for gateways to connect to end nodes further away due to higher reception sensitivity. Table 1 provides the characteristics of the LoRaWAN technology.

### 2.2. LoRaWAN

The LoRaWAN MAC protocol is an open source protocol standardized by the LoRa Alliance which operates above the LoRa physical layer. The LoRaWAN MAC layer specifies the MAC control process which allows data transmission between several end nodes and gateways. The MAC protocol provides LoRa end nodes with channel access, ADR, security and energy saving services [19]. The deployment of thousands of end nodes requires extensive access to improve simultaneous transmissions and circumvent packet collisions. The LoRaWAN MAC uses Aloha to coordinate the links, dividing airtime among end nodes to handle packet collisions. The Aloha MAC permits end nodes to forward data packets once they wake up and in the event of any collisions the exponential back-off is applied. The LoRaWAN protocol is responsible for device class allocation. The LoRa specification defines three device classes that the end nodes must operate in, Class A, Class B and Class C. The end node initiates Class A communication which is fully asynchronous. The UL message can be transmitted at any instant, followed by two short DL windows, providing a prospect for bi-directional communication. After an end node has sent a confirmed message, the end node expects an Acknowledgement (ACK) from the NS during the two pre-set timeslots known as “Receive Windows (RW)”. The gateway either responds with the first RW or the second RW. Unconfirmed messages from the end nodes do not receive acknowledgement from the NS. Periodic beacons are used to synchronise Class B devices to the network, which opens DL ‘ping slots’ at programmed intervals. The network is provided the capacity to transmit DL messages with a predetermined latency but results in added energy consumption in the end node. Class A and B end nodes are largely battery-powered but Class A utilises less power than Class B. Class B devices do not support Class C functionality. Class C devices, additional to the Class A structure of UL followed by two DL windows, further reduce latency on the DL by maintaining the end node receiver continuously listening for responses from the gateway. Because these devices are continually listening, they consume more energy and hence need to be mains powered. Class C devices do not support Class B functionality.

A LoRaWAN network will always use LoRa as its PHY layer. The LoRaWAN protocol is the suitable MAC layer but different MAC layer protocols can be used. LPWAN requirements such as ACKs, firmware updates, localisation, roaming, and security are all addressed in the LoRaWAN standard.

### 2.3. Transmission Parameters

LoRa end nodes are set up utilizing distinct SF, BW, CR and TP settings, resulting in several permutation possibilities. The LoRaWAN network performance vastly depends on the configuration of these parameters [20]. Determining the settings that curtail the cost of transmission energy whilst sustaining the communication performance requirements is challenging. Configuring these parameters helps to optimize the communication performance as these parameters have an effect on energy utilization in end nodes. Therefore, it is vital that the battery powered end nodes select transmission parameters that are appropriate in a LoRa network. Poor choices could cause a hundred-fold briefer end node lifetime, rendering numerous commercial applications unfeasible as a consequence [21]. Algorithms that can find each node’s optimum transmission parameter configuration are required for the network. Achieving configurations that are optimum involves investigating links with varied settings. LoRaWAN implementations employ static transmission parameter settings with high reliability.

#### 2.3.1. Spreading Factor 

SF is the chip rate divided by the symbol rate. It is the number of raw bits that can be encoded to a symbol. As SF increases the signal-to-noise ratio (SNR) increases, resulting in an increase in sensitivity and range. However, it results in an increase in the packet airtime. The expression $2^{SF}$ denotes the number of chips each symbol can hold [10]. The SF characterizes the relationship between the chip rate and the baseband data rate. LoRaWAN SF values range from 7 to 12, which implies that an SF value of 12 increases the strength of the communication signal by increasing the sensitivity of the receiver-equipment but the data rate decreases as a result. Conversely, a reduction in the SF causes the data rate to increase, but the message being forwarded requires a higher TP to be properly decoded at the receiver. When the signal is weak, LoRa devices use a higher SF and using a higher SF implies a longer ToA. The distance from the gateway also affects the SF. The further away the end node is from the gateway the weaker the signal and therefore the higher the SF.

#### 2.3.2. Bandwidth

In terms of LoRa modulation, bandwidth is a very crucial parameter. A LoRa symbol comprises of $2^{SF}$ chirps, that spread in the whole frequency domain. A chirp is a signal wherein the frequency increases, called an up-chirp or decreases, known as a down-chirp. The LoRa symbol begins with a set of up-chirps whose frequency increases with time. When it reaches the maximum frequency, it skips back to its lowest frequency and starts over. The down-chirp is the inverse of the up-chirp which starts at the maximum frequency and decreases with time. When it reaches the minimum frequency, it skips back to the maximum frequency and the cycle starts over. BW is a range of frequencies within a given transmission band [22]. High values of BW give higher data rates which implies a shorter ToA. This results in reduced sensitivity because of the additional noise that is integrated. A lower BW produces better sensitivity but achieves lowered data rates. Data transmission occurs at a chip rate corresponding to the BW where a BW of 125 kHz corresponds to a chip rate of 125 kilo chips per second (kcps). While the BW could be selected ranging between 7.8 kHz and 500 kHz, a standard LoRaWAN functions at either 500 kHz, 250 kHz, or 125 kHz (BW500, BW250 and BW125) according to the regional parameters [9].

#### 2.3.3. Coding Rate

LoRa uses FEC error coding to improve the robustness of the wireless connection. This type of error coding results in additional bits within the LoRa physical layer payload which is controlled by the CR parameter. The LoRa modem uses CR to provide increased protection against bursts of interference and decoding errors. LoRa permits CR settings to be either 4/5, 4/6, 4/7 or 4/8. Setting a high CR value implies that there are more error correction bits which provide better protection for the transmitted data. However, on the downside, it increases ToA which in turn decreases battery life. Receivers which vary CR and hold SF and BW constant, can still communicate between them by using an explicit header, since the CR of the payload resides in the packet header, that is encoded at CR 4/5 by default [17].

#### 2.3.4. Transmission Power

In LoRaWAN networks, the power essential for the transmission of a data packet is adjustable as appropriate. Lowering the transmission power will save the battery but shorten the signal range and vice versa. The LoRa radio the TP is adjustable from −4 dBm to 20 dBm in notches of 1 dB. However, in real life deployments, the TP range is commonly restricted to between 2 dBm and 20 dBm because of the hardware limitations. Additionally, if the TP levels greater than 17 dBm are experienced, only a duty cycle of 1% can be utilised [21].

### 2.4. Adaptive Data Rate

An essential LoRaWAN feature is the ADR scheme which seeks to minimize battery usage and maximize throughput by altering the data rate and TP for each end node in the LoRa network. Data rate adaptation in a LoRaWAN allows easy scalability of the network by the addition of gateways. Furthermore, the use of ADR significantly increases the capacity of such a network, since the data packets that are transmitted using different SFs are orthogonal and can be transmitted concurrently [23]. An ADR scheme was developed into LoRaWAN to be able to manage the end nodes’ transmission parameters to improve the packet delivery ratio (PDR). The ADR controls transmission parameter settings for the UL data from the end node to the gateway. The ADR algorithm is responsible for managing the data rate and transmission power of end nodes based on the link budget estimation in the UL message and the maximum SNR required for accurately decoding data packets at the existing data rate. In the case of fixed end nodes, the NS manages the ADR depending on the history of the UL packets received, referred to as “Network-managed ADR or Static ADR”. The network based ADR approach does not work for mobile end nodes because channel attenuation which occurs as the device moves. Where mobile end nodes are concerned, ADR is performed “blindly” on the end node side known as “Blind ADR”. LoRaWAN networks employ adaptive modulation techniques with multiple channel multiple modem transceivers in the gateway to receive multiple messages from the channels. Each specific signal uses a unique SF, with orthogonal separation provided by the spread spectrum. This technique presents advantages in data rate management [24]. LoRaWAN’s ADR scheme dynamically adapts the transmission parameters aiming to prolong battery life and maximize throughput. This is done by varying the data rate and TP for each end node in the LoRa network. ADR improves the data rate, ToA, and energy utilization. In LoRaWAN, varying the SF adjusts the data rate of the end nodes, thus optimizing the throughput. Nevertheless, the ADR must be utilized cautiously since the collision probability, that directly influences throughput, is affected by the change in SF. The ADR algorithm was established for stationary end nodes and stable radio channel environments [18]. The ADR scheme bases its choice of data rate on the past performance of each end node. The LoRaWAN MAC layer contains four different commands for the ADR shown in Table 2.

Because end nodes have limited battery capacity, the performance of LoRaWAN networks is affected by power consumption. The fact that the end nodes must accommodate specific data rates further compounds the power limitation challenge because the SNR levels must be above certain thresholds as well as the power levels. Additionally, the end nodes have to respond to the channel conditions in the network. This means end nodes must have the ability to regulate the transmission rates and power levels appropriately [25]. A LoRa gateway can listen for UL messages simultaneously on every SF and BW permutation, whilst the end nodes are capable only of eavesdropping on a single fixed SF and BW sequence successively. An end node may use any set of transmission parameters to communicate with the gateway without handshaking. Message transmission from gateways to end nodes occurs on a programmable offset from the UL data rate in the first RW, and typically with the highly robust setting, the lowest data rate in the second RW. 

An end node notifies the gateway that it requires the use of ADR by configuring the ADR bit in the frame header. Once ADR is configured, the NS uses LinkADRReq, the MAC command that controls the end node’s data rate and TP. The end node will respond with the LinkADRAns command to indicate acceptance or rejection of the new settings. The ADR algorithm comprises of an acknowledgement system which is devised to permit end nodes to intermittently verify that the NS received the UL message. If an ACK message is not received by the end node, the end node will switch to a lower data rate in an attempt to regain connectivity. The permutations of the transmission parameters produce a potential 6720 potential transmission settings of which the LinkADRReq command can only select from a subset of eight data rate settings and six transmission power settings [18]. Even though LoRaWAN stipulates a transmission parameter signalling scheme via the LinkADRReq command, there is no description available of how the communication should be handled. The specification does not state how the NS should instruct end nodes concerning adapting the data rate, when to change a setting, or the order in which the settings should be changed [22]. The NS is left with the responsibility to implement ADR. The end nodes also have the capability of managing the ADR transmission parameters using the ADR system that is nested on the end node side. This means that, the ADR scheme can run asynchronously at the NS side and the end node side.

**Figure 2:**
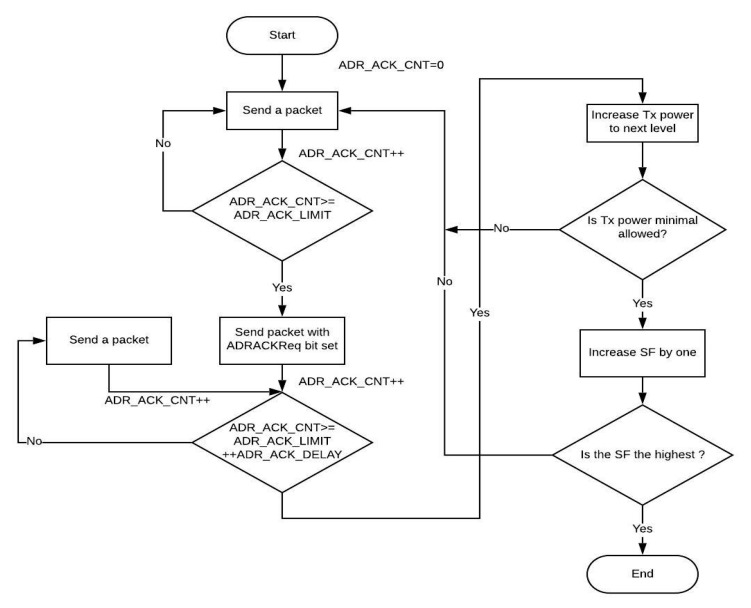
Adaptive data rate flow [26]. SF: Spreading Factor.

As stated in the LoRaWAN standard, there are two parameters that have been specified, namely ADR_ACK_LIMIT and ADR_ACK_DELAY. The default values for these parameters have been set to 64 and 32, respectively. For every UL packet that an end node transmits, ADR_ACK_CNT counter is increased by one. Once the ADR_ACK_CNT becomes equal to ADR_ACK_LIMIT without any DL response, the end node sets the ADRACKReq bit and waits for an ACK from the gateway for the subsequent ADR_ACK_DELAY UL packets. In the absence of an ACK ahead of ADR_ACK_DELAY UL message, the end node decreases the data rate, attempting to re-establish network connectivity. In accordance with the latest release, end nodes initially increase TP to secure connectivity. If that is inadequate, the end nodes then reduce the data rate as an element of the subsequent stage [26]. Figure 2 outlines the flow of the ADR scheme executed at the end node.

## 3. Review of Adaptive Data Rate Algorithms

Having an exceptional ADR scheme gives vendors a competitive edge, as such they maintain their applications confidential. Thankfully, there is an open source network manager that made its ADR algorithm publicly available—The Things Network [27]. This algorithm is based on Semtech’s recommended algorithm for rate adaptation. The LoRaWAN ADR is a scheme used to optimise the network’s data rates, ToA and energy consumption. To maximize battery life of the end nodes and global system capacity, the LoRaWAN NS controls the data rate and TP for all the end nodes independently through an ADR scheme. Several data rate adaptation schemes have been propositioned in literature that attempt to enhance communication performance. In literature ADR scheme has been modified and implemented to meet different objectives in the LoRaWAN targeting network performance metrics. We review the body of work that employs ADR to improve metrics such as scalability, throughput, channel access, received signal strength (RSS) and energy efficiency.

### 3.1. Scalability

In LoRaWAN networks, ADR control is enabled to keep network connectivity by providing numerous data transmission rates. The condition of the RF connection is determined through the receive status of the ACK packet in LoRaWAN through link errors. This, however, does not indicate a congested network but may result in inefficiencies in transmitting data due to long transmission delays. Since congestion is not considered a connectivity problem, decreasing the data rate by substituting the modulation system is inappropriate. In [28] the authors developed an ADR scheme that attempts to preclude extraneous data rate management using logistic regression. The proposed scheme recognises the congestion levels of the network by learning and then applying the result to control the data rate. The method uses data rate, RSS, and number of connections at the gateway as characteristics for learning. Evaluation of these attributes provides the congestion estimation. When the congestion is projected, the proposed algorithm revises the back-off time rather than reducing the data rate, adjusting latency instead of reducing throughput. This results in an improvement in the accuracy of data rate control and network efficiency. The NS performs the learning and delivers the results to the end nodes to predict congestion. The computation for learning is done in a centralised machine. The strength of this approach is that it considers the level of congestion in the network unlike the legacy ADR scheme. The drawback of this approach is that the process demands an ACK DL message for each transmission. Because DL traffic has a negative effect on UL throughput, the PDR decreases as a result [29]. Future research could look at studying distributed learning of the end nodes. Other methods of optimization could be applied for predicting congestion.

Framework for LoRa (FLoRa) was developed in [30], wherein the authors dynamically manage link parameters for scalable and efficient network operations. The authors developed a non-proprietary scheme for end-to-end LoRa simulation in OMNeT++. They considered a distribution of SFs on the average SNR values from several uplink packets received at the gateway as opposed to the highest SNR used in the standard ADR scheme. They use comprehensive simulations to demonstrate that ADR effectively increases the PDR, maintaining low energy utilization under stable RF conditions. Their results showed that adjusting channel conditions had a severe effect on the performance of the ADR scheme, hence the modification of the link quality indicator, and the introduction of a transmission policy to increment TP at the end nodes. The proposed ADR scheme achieved at least thirty percent better PDR compared to the standard ADR scheme in the cases of moderate variable channel conditions. The proposed scheme showed that appropriately configuring SFs and TPs could boost the network capacity and reduce energy utilization, based on the overall knowledge of the network. It implements an ADR algorithm wherein end nodes could dynamically update their SF and TP. Although the proposed algorithm shows significant increase in reliability and energy efficiency, there is a drawback. Waiting for twenty frames in order to adjust the scheme may be too long. For dense networks, link-based adaptation is inadequate. Future work could consider incorporating collision probability and the distribution of parameters in the network. Balancing the link budget for every link and PDR of the entire network could further improve scalability.

Reynders et al. [31] proposed a MAC layer protocol RS-LoRa which distributes SFs and TPs to reduce the capture effect and inter-SF collisions and improve network reliability and scalability. They introduced a two-step lightweight scheduling method that divides end nodes into clusters, where identical TPs are applied in each cluster to diminish the capture effect. First, the end nodes get recommended by the gateway’s coarse-grained scheduling to apply distinct SFs and RSS to allow concurrent transmission, therefore reducing packet collisions. The gateway broadcasts beacons to the end nodes before the nodes can send any packets. Depending on the coarse-grained information supplied by the gateway, the end nodes select the parameter combination and channel that best suits the nodes. Using this approach, reliability of the network is improved by reducing packet error rate (PER) up to twenty percent compared to standard LoRaWAN using NS-3 simulation. Improved network reliability improves network scalability which in turn contributes to an improvement in the throughput. Network performance improves significantly when there are many end nodes in the network to the tune of one thousand, for example. Although the light-scheduling approach improves reliability and scalability, there is an introduction of additional energy consumption as the end nodes need to listen for the beacon from the gateway before sending a packet. The approach does not eliminate packet collisions entirely because uplink messages could still collide with beacons from other gateways. Because the approach uses Aloha, it also means collisions cannot be eliminated. Future work could look at using a different MAC protocol to improve PER.

The authors in [32] modified the standard ADR algorithm to produce an expansion of the network scalability, fairness between nodes, PDR and robustness to dynamic channel conditions. The algorithm facilitates the system to optimize SF and TP for each end node with the purpose of increasing reliability of communication and optimizing energy usage at the end nodes. The authors recommend varying data rates before increasing TP, averaging of SNR history, and accounting for hysteresis. With empirical evaluation of the relationship between PER and SNR the authors find a channel model which they subsequently applied in MATLAB simulations. Certain alterations of the algorithm, enable the achievement of improved error performance in rough channels resulting in the reduction in the amount of retransmissions and DL commands from the NS. The algorithm decreases the data rate before increasing TP whenever a given fixed threshold is exceeded. They introduce variable hysteresis into the algorithm which mitigates against the effect of collisions and duty cycle limitations. The proposed algorithm reduces the number of data messages in the UL as well as the MAC command messages in the DL and achieves superior error performance in poor condition channels. This enables the extension of the network range. The drawback of this algorithm is that the simulation model used does not consider large complex networks.

A model that optimizes attenuation and collisions in order to optimally allocate the SF was proposed in [33]. The model aims to optimize the quantity of end nodes distributed in the network whilst assigning SFs that maximize transmission quality. They consider physical capture and imperfect SF orthogonality whilst ensuring a specified probability of successful transmission to every end node within the network. The approach considers an authentic propagation model which considers physical capture that could surface at the gateways. They consider inter-SF and intra-SF interference for each node as a potential for packet collisions. However, it assumes that the density of the end nodes within the gateway range is uniform, which is not realistic. End nodes covering areas of several square kilometres would be highly non-uniform. This mathematical model needs to be validated by simulation to quantify the gains of appropriate SF allocations regarding PDR in a realistic environment.

Bor et al. proposed a dynamic transmission scheme and made the network denser by adding more gateways. They experimentally observed the capture effect of LoRa, that, where signals with the same SF are transmitted simultaneously, the strongest signal suppresses the weaker signals when the difference in power is adequately large. They developed a LoRa simulator (LoRaSim) and analysed the LoRa scalability threshold in fixed settings. They modelled the capacity of such networks by introducing the Data Extraction Rate (DER) metric, modelling uplink behaviour and proposed a mathematical model for transmission range, dependent on the experimental data gathered. They concluded that the network with a single gateway and moderate transmission parameters did not scale well, whilst those with dynamic adaptation of transmission parameters or multiple gateways tended to scale better. They found that network scalability increases when the parameters configuration minimizes the ToA. This model, however, overestimates link attenuation of LoRa signals in free space. The model needs on-site measurements which are difficult to obtain since most of the area of coverage comprises of conventional connections, classified as connections with dynamic temporal link attributes. Although the use of multiple gateways outperforms existing results, optical placement of the gateways consistent with the category of application would further improve the performance.

Finnegan et al. [34] presented significant improvements aimed at the end node and gateway that decrease the convergence time for LoRa nodes to attain optimum data rate. They extended the LoRaWAN component in NS-3 by including the ADR, thus allowing the simulation of lifelike LoRaWAN networks by implementing novel improvements in this module. The simulations demonstrate a significant decrease in convergence time for the end nodes due to the modifications. This leads to an increase in global PDR for the network in a dynamic network environment. The authors presented an evaluation of the behaviour of the standard ADR scheme and proposed a new variant of the scheme with improvements that enhance performance in all cases whilst maintaining the ability to be efficiently integrated into an existing LoRaWAN network.

The authors in [35] employed SF allocation as a tool to increase LoRa network capacity. They defined an optimization problem for SF allocation by maximizing Packet Success Probability (PSP) to maximise end node connectivity. In their scheme, the authors consider both inter- and intra-SF interference and when assigning the SF allocation. The interference considerations are incorporated through the capture effect when the signal of interest maintains a Signal-to-Interference (SIR) which surpasses a certain threshold. The propositioned approach controls the SF distribution dependent on the assignment distances resulting from the solution of the optimization problem instead of the coverage distances derived from the physical layer LoRa sensitivities. The authors use stochastic geometry to determine the average system PSP. They assign SFs to end nodes with two considerations: (a) the received power from the end node must surpass the receiver sensitivity threshold for the allotted SF, and (b) the SIR for the end node must similarly eclipse the accurately decoded SIR threshold for that SF. Although the complexity is high for multiple variables, the global optimization solver can be used to solve the problem.

A method to improve the capacity of Mesh LoRa networks using network clustering which is based on SF was proposed in [36]. They used a Tree-Based SF Clustering Algorithm (TSCA) which allocates nodes to numerous subnets. They use the LoRa transmission parameter selection to create mesh networks. The approach is rooted at the gateway in which every tree is a subnet with a different SF to enable simultaneous transmissions. TSCA balances the traffic load to avoid bottlenecks at the subnet using a SF capacity estimation considering the number of nodes, data rates and hop count of the subnets. Minimizing the number of hops and the delay is the objective, since higher SF values result in more airtime, leading to an increase in the end-to-end delay. According to the authors, their results showed an improved performance in contrast with the SF allocation in a single-hop LoRaWAN network. Future research could involve a faster approach in predicting connectivity that would utilise the quickest data rate for the connectivity prediction of all the SFs.

### 3.2. Throughput

ADR can be used to improve network throughput by improving DER. [37] propose two algorithms of incremental complexity EXPLoRa-SF and EXPLoRa-AT. EXPLoRa-SF is a heuristic which attempts to equally distribute SFs to the end nodes in the gateway’s radio range, only restricted by their Received Signal Strength Indicator (RSSI) values and relevant thresholds. EXPLoRa-AT on the other hand, propagates impartial allotment of the ToA between the end nodes in the network. The proposed scheme does not configure the SF on the basis of distance and received power measurement, but instead considers the quantity of linked end nodes, enabling the equalisation of the ToA of the packets in each SF. EXPLoRa-AT guarantees equalisation of the RF channel utilization by the end nodes by leveraging the use of multiple SFs in order to have orthogonal sub-channels. The two algorithms combine SF orthogonality and radio range visibility to expand the density of end nodes with concurrent transmission in the grid, reducing collisions, improving DER and ultimately improving throughput. The ordered waterfalling technique is used for even distribution of channel load between the end nodes in the system. For dense networks, the algorithms substantially improve data rates and robustness. Simulation of EXPLoRa-SF and EXPLoRa-AT in “LoRaSim” has a superior performance compared to the standard ADR. These two algorithms are implemented using one gateway. The drawback of this approach is that it is designed under the assumption that end nodes transmit with the uniform data rate and payload. The work in [38] attempts to address this drawback. There are circumstances where the assigned SF in EXPLoRa-SF does not adhere the restrictions stipulated in LoRaWAN that degrade performance. Additionally, the proposed approach in [37] does not support implementation with multiple gateways which is an open gap for future research.

A contention-aware ADR scheme, developed by [39] traces the distribution of end nodes for every SF and seeks to expand the number of gadgets utilizing low SFs. The authors attempt optimization of throughput in networks where the same SF can be used by a huge number of end nodes. The scheme uses constrained optimization and defines the total throughput as an objective function with respect to the quantity of end nodes utilizing specified SFs. The authors develop the theoretic optimum throughput which could be accomplished by changing only the data rate of the end nodes. They adopt the gradient projection method to solve the constrained optimization problem, which enables the gateway to effectively obtain the optimum configuration subject to the constraints, irrespective of the number of end nodes implemented in the system. When numerous end nodes have analogous link quality, specifically in relation to the partial use of the SF, the propositioned approach achieves significantly better throughput than the existing scheme due to the load balancing effect. The strength of the approach is factoring in the contention issue in optimising ADR. Although this approach improves throughput, the transmission success ratio of the end nodes declines, so if reliability is more important than throughput in an application, this approach would not be sufficient. For future research, a more comprehensive evaluation and efficient solution for the SF update via DL is an area that is open. An optimization technique can be developed that considers the transmission success ratio at the same time, thus extending the solution to the multi-objective optimization problem.

A Fair Adaptive Data Rate algorithm (FADR) that calculates data rate and TP assignment with the purpose of achieving fairness in data rate and reducing collisions between the end nodes is propositioned in [40]. The algorithm uses RSSI values in its computations while deciding SF and TP allocations to maximize DER among all end nodes. The authors propose a “region concept” to assign SFs depending on the RSSI, and within the regions they are allotted SFs corresponding to the proportions provided. The fairness of resource distribution is accomplished by the proposed power allocation scheme, which aims to optimize the RSSI of the end nodes, allocating low TP to the end nodes with a weak signal and high TP to end nodes with a weak signal. They achieve uniform DER for all the end nodes notwithstanding the distance from the gateway and maintain the end node’s lifespan by applying low TP levels reducing power consumption by twenty two percent compared to standard ADR. The authors showed that by incrementing the number of end nodes in the network, the fairness index between end nodes decreases. This would favour communication from end nodes nearer to the gateway in comparison with those further away. This approach is therefore only feasible in extremely small networks whose end nodes are positioned near the gateway. Using different propagation models in the simulation is worth investigating.

A mathematical model whose numerical analysis results in a closed form formula that maximizes throughput whilst complying with transmission duty cycle (TDC) regulations was proposed in [41]. Their model mitigates against strict restrictions imposed on duty cycle of ISM bands in some regions and improves transmission and power efficiency in the end nodes. The approach presents two meta-heuristics to solve an optimization problem by computing the transmission policy. An optimal transmission policy means optimal selection of BW, SR, CR and TP. By considering band utilisation and power utilisation efficiency together, the highest achievable throughput of end nodes is calculated and optimized regarding a series of possible transmission parameters, hence achieving the optimal transmission policy. The authors resolve the convolution of LoRaWAN networks by modelling the core status of end nodes applying the Markov model. In so doing, they develop a closed-form formula for node performance which is optimized mathematically with classic maximization algorithms. This method increases performance by over thirty three percent compared to the standard ADR scheme and is indicated to be able to operate in hardware constrained IoT devices. The strength of this approach is that the model is based on an optimal policy derivation theory, which gives the approach the ability to statistically predict the performance of the end nodes in the LoRaWAN network. The drawback is that it is a complex combinatorial optimization problem which cannot be solved directly. Future research could investigate the use of a simpler optimization approach.

Reinforcement Learning (RL) based approach is used in [42] wherein they derive efficient ways of disseminating updated transmission configurations to maximize throughput while mitigating against TDC limitations. The authors mathematically model the average throughput per end node as a function of the packet generation behaviour of the end nodes such that the optimal transmission parameters are obtainable. They use the RL-based algorithm to update the configuration of individual end nodes to maximize the accumulated throughput per end node. They then analyse the behaviour of the LoRa nodes focusing on when and how packet collision may occur. Centred on this analysis, they mathematically depict the performance of LoRaWAN as a function of the transmission characteristics of the constituent nodes. The results show a significant improvement in throughput per node compared to standard ADR algorithm. The strength of this approach is that it takes into consideration the fact that IoT networks are heterogenous, that send packets at different packet rates and varying payloads. In the model they consider randomly distributed end nodes and the capture effect. Instead of just maximising LoRa performance, they update transmission parameters of the overall network configurations. This approach improves throughput but did not look into its effect on power consumption. Further study on how this approach can be used to aggregate transmission settings from different gateways in a single updating packet in a LoRa system with multiple gateways is open for research.

Channel attenuation and data collision are classified as reasons for packet loss in [42]. The authors formulated an ADR selection scheme with enhanced loss differentiation which selects a reasonable data rate that maximizes throughput. The solution comprises three parts: prediction of channel attenuation, data collision probability and a data rate controller. They establish a channel attenuation model which they use to determine packet loss probability by channel attenuation. They then find the probability of data collision due to interference. A simulation model is established to investigate the correlation between the number of end nodes and the packet loss ratio (PLR) in the LoRa network using one gateway. They use MATLAB simulation to analyse and validate the performance of the technique. The proposed scheme showed reduced packet loss and improved DER compared to the standard ADR scheme and EXPLoRa. In this approach the authors only considered a situation where the nodes report periodically. Future work could look at event-driven applications as a consideration.

A concept of network slicing was introduced in [43]. The LoRaWAN network is partitioned into several virtual networks, termed network slices, to efficiently assign network resources to support specific QoS requirements for every slice. Within the IoT, every end node demands specific QoS requirements regarding delay and reliability determined by the type of IoT application being run. The authors use various network slicing strategies with different SF distributions to evaluate the network performance and optimise SF allocation for each slice. They propose an adaptive dynamic inter-slicing algorithm wherein the BW is reserved on each LoRaWAN gateway using Maximum Likelihood Estimation (MLE). They then improve that algorithm by considering each gateway individually, reserving its BW after utilising MLE on the end nodes within its range (intra-slicing algorithm). They compared the two adaptive dynamic slicing propositions to a standard fixed slicing strategy in which the gateway’s BW is reserved uniformly among all the slices. Their results indicated an improvement in the optimisation of ADR due to an efficient coordination of resources. The strength of this approach is the provision of isolation of end nodes and its ability to maximize the efficiency of resource allocation in each LoRaWAN slice. The drawback is that energy consumption in the adaptive dynamic network slicing algorithm is increased compared to the static and dynamic configuration. The authors extended this work in [44] by taking into consideration some smart city applications representative of different QoS classifications and used a slice-based SF and TP configuration optimization. They proposed a new slicing optimization method called TOPG that is formulated on the Technique for Order of Preference by Similarity to Ideal Solution (TOPSIS) and Geometric Mean Method (GMM). Their suggested scheme sets the LoRa SF and TP parameters efficiently to improve the performance of every slice in terms of QoS, reliability and energy utilization. The results showed that TOPG outperforms static and dynamic configuration strategies, highlighting its effectiveness in the provision dynamic slice-based configurations and improving the performance of LoRa slices with regard to reliability and the percentage of end nodes that fulfilled their throughput and delay requirements.

The joint SF assignment and transmit power allocation algorithm has been investigated in [45]. They consider both co-SF and inter-SF interferences for the improvement of throughput fairness. In theory, there is a dramatic decrease in performance levels when orthogonality conditions are not met under the same SF conditions and communication channel. The authors formulated a joint SF and TP distribution problem to maximize the minimum UL throughput of the end nodes, dependent on co-SF and inter-SF interferences and power constraints. They based their strategy of SF allocation on the matching theory. Once the end nodes have been allocated SFs, optimization of the power distribution parameters is performed so as to maximize the minimal throughput achieved on each SF. The authors addressed the intractability of the joint SF and power distribution problem by splitting it into two subproblems: SF allocation under fixed TP, and TP distribution under fixed SFs. They use both linear and quadratic approximation to make the non-linear inequalities in the feasibility problem tractable. Their simulation results showed that in spite of severe co-SF and inter-SF interferences, the propositioned algorithms outperform standard algorithms, jointly with regards to minimum end node data rates, user fairness, and average end node throughput. The two propositioned linear and quadratic approximation methods to tackle the non-linear feasibility problem for TP distribution produced efficient TP solutions, resulting in significant energy conservation, whilst further improving minimal throughput and user fairness.

### 3.3. Energy Efficiency

Authors in [20] evaluated the performance of LoRaWAN under different scenarios using computer simulation and testbeds. They investigate the network performance regarding PDR, mean energy usage per transmission and mean energy utilization due to packet collision per node. They show that the LoRa network performance is enormously reliant on to the configuration of SF, CR, and BW. The experiments show the access strategy of pure Aloha has limitations which affect performance. The short-coming could be mitigated by the randomization of BW selection and node-specific optimization to maximize data rate in relation to its channel conditions and contention severity. They conduct several experiments, the first which considers robustness and uplink data rate which they evaluate with and without the capture effect. In the second experiment they incorporate multiple frequency randomisation to minimize the collisions. In this experiment, they adopted the slowest case from Experiment one. The results show that the frequency randomization that was introduced is capable of increasing the PDR of the slowest case. In the final experiment they contrast two simple optimizations. The first optimization, Opt1, sets the parameters in every end node so as to minimizes ToA, which is determined by its distance from the gateway. The second optimization, Opt2, sets the parameters in every end node so that it minimizes its ToA and energy consumption. In other words, Opt2 attempts to minimize the energy utilization of the solution attained by the Opt1. The results show that optimization can yield a higher PDR and lower energy consumption. Interference from other ISM frequency users was not considered in this approach but could be a crucial factor which affects the performance. This could be interesting for future research. It could also be noteworthy to examine the effect of the different classes of the end nodes and their DL transmissions including the presence of multiple gateways in the vicinity.

In [46] the authors aim to optimally transmit data with a high PDR whilst maintaining a low energy utilization. They introduce integer linear programming models to establish the optimum allocation of the parameters by considering the distribution of the end nodes and the impact of transmissions from the surrounding end nodes. They split the optimization into two phases. First, they optimize the SF such that the collisions in the SF with the most traffic are marginal, and the collisions in every SF is balanced for all gateways to guarantee reliable communication within the network. As the problems in consideration are non-linear, they are converted into tractable integer linear programming models. Secondly, they optimize the TP to minimize the energy consumption in the network. After obtaining the optimum configuration, they analyse the solutions by means of extensive network simulations and compare them to different state of the art algorithms. The results illustrate that the optimal configurations consistently perform better, achieving a greater PDR and nominal energy utilization across different scenarios. The approach can guarantee that a significant proportion of end nodes communicates reliably with a high PDR. All the network end nodes share the improved PDR, thus guaranteeing an unbiased distribution of RF resources to all the end nodes. The strength of this approach is that the models are general, thus allowing network configuration with single or multiple gateways, along with different spatial configurations of LoRa devices. Commercially available solvers can be used to solve the optimization model within a short space of time. This means that even for huge and dense networks with thousands of devices, the proposed model can be readily employed by service providers to establish the optimum setting of the LoRa parameters. The dynamic reconfiguration of the LoRa parameters could be an open area for research. More accurate path loss parameters could be estimated using linear regression on measured data instead of sourced data [47] used in the experiments. The deployment environment of the network highly affects the actual path loss parameters. 

An ADR scheme that efficiently optimizes the PER fairness within a LoRaWAN cell was developed in [48]. The authors achieved this by optimising the TP and SF for every end node whilst circumventing near-far problems by allotting outlying end nodes to different channels. End nodes are arranged by their pathloss and divided into uniform clusters whose number is equivalent to the number of available channels. Every cluster is allocated a distinct channel and inside the clusters the proportion of end nodes employing the SFs is relative to s/2^s^, corresponding to the solution of the optimization problem to minimize the maximal collision probability among all the SFs. The algorithm calculates the optimal SF allocation to utilize in order to minimize the collision probability. The scheme optimally assigns SFs and distinct power levels to nodes within a LoRaWAN cell such that the signals do not interfere with each other. This scheme improves the PER for end nodes further from the gateway. Simulations of this approach implemented in NS-3 demonstrate that the PER can be reduced by up to fifty percent for end nodes further away from the gateway in a moderate contention scenario. The overall network PER is reduced by 42%. The approach reduces operating expenditure by reducing energy consumption providing wide network coverage which in turn reduces the number of gateways. The scheme uses uniform distribution of the end nodes around the gateway and all the end nodes can use all SFs and TPs, that is, all end nodes in the network are capable of reaching the gateway with each SF and each TP configuration. This does not function in actual networks where specific end devices can only utilise a subset of the configuration parameters determined by the distance from the gateway. Further study could investigate randomly distributed end nodes. They consider unacknowledged traffic in this work.

Modulation and Coding Schemes (MCS) affect the transmission duration of the data frames. Data delivery time is dependent on network performance which impacts on energy consumption. For example, [49] considers failures in attempts to transmit triggered by arbitrary noise in the channel. The authors develop a precise analytical model of the data delivery time, dependent on the network set up. They show that their model can be used in the MCS (SF, BW, CR) the election process to fulfil heterogenous QoS requirements of different types of traffic. They presented a precise mathematical model of transmitting data which allows estimating performance indices such as PLR payload and delivery time distribution. The strength of this approach is that it considers heterogenous network with various types of traffic and QoS requirements. Furthermore, the proposed model allocates MCSs such that QoS requirements are fulfilled. The algorithm has a drawback in that it does not consider inter-SF interference which is vital for huge network loads. The model can be improved to not only improve the packet loss ratio in the network, but in addition the PLR distribution and to take into account the non-orthogonality of SFs in the model.

In [50] the authors develop an approach that improves the QoS of the LoRaWAN network by reducing data collisions and energy consumption, and increasing the DER. They use the Mixed Integer Linear Programming (MILP) optimization method to generate optimum settings for SF and Carrier Frequecy (CF) parameters. LoRaSim simulation is employed to demonstrate the effectiveness of the approach for different network scales. For their model, the authors assumed that BW and CR are fixed in computing ToA while they varied CF and SF to maximize the success probability. Three evaluation metrics were used to assess the network performance, namely, DER, number of collisions and network energy consumption. Simulation results demonstrated that MILP optimized the assignment of SF and CF pairs with over six percent increase in DER compared to the standard LoRaWAN ADR and number of collisions thirteen times smaller. The network energy consumption became almost three times lower than the equal-distribution and random dynamic allocation strategies. The strength of their approach is that it has backward compatibility with the standard ADR scheme, implying they are implementable in off-the-shelf LoRa devices. Future work could entail extending the optimization approach to longer transmission distances and a bigger number of gateways. 

The authors in [51] present a performance improvement technique through SF distributions for LoRa networks. They formulate the optimization problem for the SF distribution to maximize the packet reception probability (PRP) under a constraint for the mean energy utilization of each end node. This makes provision for the network performance to improve under the constraint for each end node by solving this optimization problem. The authors develop a technique to solve the formulated problem based on a distributed genetic algorithm, which is a metaheuristics technique. The technique improves the network performance by assigning the SFs to end nodes under the constraint for the average energy consumption of each end node. The assumption is that the end nodes are static and their number in the network does not change. The PRP is derived while considering the imperfect orthogonality of the SF in LoRa networks. Their results showed that the PRP performance of their proposed technique is superior and uses less average energy utilisation for all end nodes compared to the existing schemes. Future work could involve energy consumption per node rather than an average for all the end nodes.

## 4. Comparison and Discussion

In this paper, we performed a comprehensive analysis of solutions that have been developed to optimize ADR algorithms. The proposed optimization techniques address specific challenges such as scalability [28,30,31,32,33,34,35,36], throughput [37,38,39,40,41,42,43,44,45] and energy efficiency [20,46,47,48,49,50,51]. Our analysis distinguished the approaches used and highlighted the challenges and performance in the studies considered. Table 3 shows a summary of the comparison of the reviewed literature. The analysis of the literature shows that existing ADR schemes use different algorithms with different computational complexities to optimize the data rate, depending on the different goals such as RSS, congestion, capture effect and channel contention. Computational complexity refers to the amount of resources required to run the algorithm, particularly time and memory requirements expressed as a function n→f(n), where n is the size of the input and f(n) is the worst-case complexity, or the average-case complexity. In [44] the adaptive slicing and SF-TP configuration algorithm has a constant complexity of O(1) for the static algorithm owing to its simplicity. However, the overall complexity of the proposed dynamic adaptive slicing and SF-TP algorithm and TOPG algorithm is $O\left(n^{2}\right)$. Complexity is minimised in TOPG due to the server reducing the search space to SF values that acknowledge the guaranteed bit rate threshold. The computation time is reduced without a significant effect on QoS performance. In [45] the running time of the developed SF-Allocation algorithm is upper bounded by $O\left(NM+Q^{2}+M^{2}\right)$. The complexity of the matching algorithm is not a constraint in an actual LoRaWAN because the algorithm operates on the NS whose computational capacity is expansive. In [48] the number of end nodes was capped at 1000 because of the restricted memory of the computer. All the transmission parameter needed to be broadcast to the end nodes resulting in a $O\left(n^{2}\right)$ memory consumption. In [50] the Approximation Algorithm maintains a linear complexity time O(n)=111n+57 in the worst-case. The algorithm is designed to function in the LoRaWAN Application Layer and end nodes with a time complexity that is equivalent to the ADR scheme, so that the proposed optimization algorithm does not cause any substantial computation overhead, neither in the end nodes nor in the NS. Generally, the operation of the algorithm uses less than 20 kB of memory, 4 kB on average and 20 kB being the worst-case. This is insignificant since most commercial off-the-shelf end nodes contain no less than 128 kB of flash memory [52].

The algorithms used to improve scalability in LoRaWAN were discussed in [28,30,31,32,33,34,35,36]. The strength of the approach in [28] is that it considers the level of congestion in the network unlike the legacy ADR scheme. The drawback of this approach is that the process demands an ACK DL message for each transmission. Although the proposed algorithm in [30] shows significant increase in reliability and scalability, there is a drawback. Waiting for twenty frames in order to adjust the scheme may be too long. For dense networks, link-based adaptation is inadequate. Future work could consider incorporating collision probability and the distribution of parameters in the network. Balancing the link budget for every link and PDR of the entire network could further improve scalability. In [31] although the light-scheduling approach improves reliability and scalability, there is an introduction of additional energy consumption as the end nodes need to listen for the beacon from the gateway before sending a packet. The approach does not eliminate packet collisions entirely because uplink messages could still collide with beacons from other gateways. Because the approach uses Aloha, it also means collisions cannot be eliminated. The proposed algorithm in [32] reduces the number of data messages in the UL, as well as the MAC command messages in the DL, and achieves superior error performance in poor condition channels. This enables the extension of the network range. The drawback of this algorithm is that the simulation model used does not consider large complex networks. The use of a Tree-Based SF Clustering Algorithm (TSCA), which allocates nodes to numerous subnets in [36], vastly improves scalability as is the characteristic of mesh networks. In our opinion, the algorithm in [35] is the best approach in improving scalability as the proposed solution outperforms the equal-interval and equal-area based SF distribution schemes in terms of average network PSP.

ADR schemes that maximize throughput maximizing solutions for the ADR scheme were discussed in [37,38,39,40,41,42,43,44,45]. The ordered waterfalling technique used in [37] for even distribution of channel load between the end nodes in the system by equally distributing SFs and then channel utilization. This algorithm is designed under the assumption that end nodes transmit with the uniform data rate and payload which is not practical. When we compare it to [39] which uses the gradient projection method we find that their approach also uses the load balancing approach but factors in the contention issue in optimising ADR making it a better scheme in maximizing throughput. The algorithm in [40] uses all four transmission parameters to maximize the DER and attempts to fairly allocate data rates. The algorithm works with nodes close to the gateway, making the solution suitable only for small networks. The algorithm in [42] it takes into consideration the fact that IoT networks are heterogenous, that send packets at different packet rates and varying payloads. In the model they consider randomly distributed end nodes and the capture effect. Instead of just maximizing LoRa performance, they update transmission parameters of the overall network configurations. Network slicing brings an interesting dimension in ADR optimization which sets the LoRa SF and TP parameters efficiently to improve the performance of every slice in terms of QoS. In our opinion the algorithm in [45] provides the best approach for maximizing throughput, outperforming standard algorithms, jointly with regards to minimum end node data rates, user fairness, and average end node throughput. 

The challenge of conserving energy in LoRaWAN networks is a QoS in the ADR algorithms in [20,46,47,48,49,50,51]. In [20] they first optimize every end node to minimise the ToA, which is determined by its distance from the gateway, searching for optimal gateway location ensuring all nodes are connected. They then use the results of the first optimisation to do a second optimisation that minimizes energy consumption. Although the results show that optimisation can yield higher PDR and lower energy consumption, interference from other ISM frequency users was not considered in this approach, yet it could be a crucial factor which affects the performance. In [46] they also optimise two parameters, first they optimise SF allocation to ensure reliable communication and then optimise TP to minimise energy consumption in the network. The approach guarantees that a significant proportion of end nodes communicates reliably with a high PDR. All the network end nodes share the improved PDR thus guaranteeing an unbiased distribution of RF resources to all the end nodes. It is a better approach compared to [20] because the models are general, thus allowing network configuration with single or multiple gateways, along with different spatial configurations of LoRa end nodes. It is a more practical approach. In [48] The scheme uses uniform distribution of the end nodes around the gateway and all the end nodes can use all SFs and TPs, that is, all end nodes in the network are capable of reaching the gateway with each SF and each TP configuration. This does not function in actual networks where specific end devices can only utilise a subset of the configuration parameters determined by the distance from the gateway. In [49] they use an optimisation approach with three transmission parameters, SF, TP and CR, to develop an analytical model for heterogenous network with various types of traffic and QoS requirements. The algorithm has a drawback in that it does not consider inter-SF interference which is vital for huge network loads. The model can be enhanced to improve the packet loss ratio in the network and energy consumption. In [50] they optimise the ADR scheme using SF and CF to maximise the success probability while fixing BW and CR to compute ToA, improving the DER, thus conserving energy. Out of all these schemes whose objective is energy efficiency, the most promising solution in our opinion, is [51] The technique improves the network performance by assigning the SFs to end nodes under the constraint for the average energy consumption of each end node.

The study of ADR in LoRaWAN networks reveals that common objectives for the proposed algorithms are scalability, throughput, and energy efficiency. Testbeds, simulations, and mathematical models are employed to develop and evaluate ADR algorithms and schemes. The mathematical models use machine learning and mathematical optimization to optimize the ADR schemes. Various ADR algorithms draw in different metrics that influence the standard transmission parameters where others consider coverage, channel access/ contention, RSS, PDR and so on, as shown in Table 3. There are trade-offs between achieving high data rates or energy consumption and the performance metrics required. Many schemes use a single gateway in their proposed solutions as it is a simple and straight forward network. Most of the literature reviewed evaluated their ADR schemes using the simulation tools such as NS-3, MATLAB, OMNET++, FLoRa and LoRaSim. The algorithms are mostly simulation based as testbeds prove to be very expensive. 

## 5. Research Gaps and Future Direction

The research conducted showed that the ADR algorithms that have been analysed prioritize different performance metrics and hence provide various solutions. Data packet collision and transmission duty cycle are issues that are common in many of the ADR schemes. Most approaches implement their ADR schemes using a single gateway. Research gaps and future work that were identified in the literature reviewed are as follows:

### 5.1. Machine Learning 

In the solutions that use machine learning, the NS performs the learning and delivers the results to the end nodes to predict some metric being monitored, for instance network congestion, using centralised machine learning. This approach centralises the training data on one machine, the NS. This can create a bottleneck in collecting training data. Future work may include using distributed learning of the nodes rather than centralised learning. Distributed machine learning enables end nodes to collaboratively learn the prediction model whilst keeping all the training data on the end node and reducing learning error. Future work could also include the use of different optimization methods for predicting the network metric under scrutiny.

### 5.2. Transmission Policy 

Because of its implementation in the ISM license-free frequency band, LoRa deployments are bound by strict legal protocols, especially where no listen-before-talk schemes are utilized. ISM bands are regulated by the TDC to determine the maximum time the band can be occupied per hour. For example, in Europe, the ETSI TR 103,526 documents rule that, for the 868.0–868.8 MHz band, the maximum allowable TDC is one percent. This implies that, IoT devices may not occupy the ISM band for more than thirty-six seconds per hour, prohibiting the transmission of new packets when this limit is attained. Schemes that mitigate against strict restrictions imposed on duty cycle of ISM bands are an open area for research.

### 5.3. Perfect Orthogonality 

LoRa uses orthogonal SFs. These allow the network to preserve the battery life of the end nodes linked to the network by adaptive optimization of each end node’s power setting and data rate. Many ADR algorithms assume perfect orthogonality and do not consider inter-SF interference which is vital for huge network loads. Inter-SF interference decreases network performance considerably, especially for high SFs where packets have a higher ToA. The models can be improved to not only improve the packet loss ratio in the network, but in addition, the PLR distribution and to take into account the non-orthogonality of SFs in the model.

### 5.4. Homogenous End Nodes 

Majority of the proposed ADR schemes consider homogenous end devices which transmit fixed payloads. In actual deployments, IoT networks are heterogenous, that send packets at different packet rates and varying payloads. Further study can include implementing ADR schemes in networks that are heterogenous and determining the power consumption in such networks.

### 5.5. Mathematical Models 

Mathematical models are common in optimizing ADR schemes. The models help in studying the different metrics and predicting the behaviour and then solving the optimization problem. The models can solve the problem of collision and duty cycle limitations for example. However, most models consider simple single gateway networks and do not support implementation with multiple gateways. Future work would entail optimising the placement of the multiple gateways and incorporating large complex networks in the models. Additionally, some models use complex combinatorial optimization problems which cannot be solved directly. Future work would be to find simpler optimization methods.

### 5.6. Uniform Distribution of End Nodes

The assumption that an ADR scheme uses uniform distribution of the end nodes around the gateway and that all the end nodes can use all SFs and TPs suggests that all end nodes in the network are capable of reaching the gateway with every SF and every TP setting. This does not function in actual networks where specific end devices can only utilize a subset of the configuration parameters determined by the distance to the gateway. Further study could investigate randomly distributed end nodes. 

Different optimization models are implemented in the ADR schemes. Where machine learning algorithms are utilized, one could use fuzzy logic or mathematical optimization and vice versa. Algorithms that consider network congestion, packet collision probabilities, the use of multiple gateways could improve the ADR. Different propagation models, randomly distributed end nodes, packet loss ratio and non-orthogonality of SFs are issues to be considered in the models to improve performance.

## 6. Conclusions

The LoRa specification does not state the way the NS should instruct end nodes pertaining to data rate adaptation. This has resulted in a gap in terms of ADR implementation as vendors keep their implementations private. Because of this, new ADR schemes are always still developing. Considering the strength and weaknesses of existing ADR schemes paves way for more efficient and effective algorithms. This paper reviewed the ADR schemes that have been proposed in the public domain and classified the solutions. We discussed the impact of the ADR solutions on the performance of LoRaWAN networks. There are numerous ADR schemes that have been proposed in literature that used different techniques to accomplish desired optimization goals depending on the objective of the scheme responding to specific performance needs and applications. The study revealed that although the transmission parameters are standard, the methods and considerations for ADR to improve network performance are countless. We identified the gaps in literature and propose future work on ADR optimization. Although existing solutions achieved promising performances, they are not optimal, therefore there it is essential to find efficient solutions. To this end, ADR algorithms can be improved to increase the energy efficiency and performance of energy-constrained end nodes. 

## Figures and Tables

**Table 1:** Characteristics of LoRaWAN [18]. CSS: Chirp Spread Spectrum, FEC: Forward Error Correction.

Characteristic	Description
Modulation	CSS
Frequency	Sub-GHz ISM: EU868, EU430, US918, AS433
Bandwidth	125 kHz and 250 kHz
Data rate	0.3–5 Kbps
Range	5 km (urban), 20 km (rural)
Maximum payload	250 bytes
Error correction	FEC
Data transmission	Half-duplex
Topology	Star
Standardization	LoRa-Alliance

**Table 2:** LoRaWAN Adaptive Data Rate (ADR) Commands. NS: Network Server, UL: uplink.

Command	Description
ADR	End node sets this bit requesting the gateway to control its data rate.
ADR = 1	NS will control the end nodes data rate
ADR = 0	NS will not control the end nodes data rate
ADRACKReq	Allows end nodes to periodically receive confirmation that NS is receiving UL messages
ADRACKReq = 1	NS should respond to confirm receipt of UL data
ADRACKReq = 0	Confirmation of receipt of UL data not required.
LinkADRReq	Transmitted by NS to request end node to change its transmit parameters
LinkADRAns	Transmitted by nodes in response to LinkADRReq command
LinkADRAns = 1	Transmit parameters successfully set
LinkADRAns = 0	Command is discarded

**Table 3:** Comparison of Adaptive Data Rate Techniques.

Ref	Objective	Optimization Approach	Testbed	Simulation	Mathematical Model	Channel Contention	RSS	PDR	Coverage	Computational Complexity
[20]	Energy Efficiency	Machine Learning	✓	✓		✓		✓		
[28]	Scalability	Machine Learning	✓	✓	Logistic Regression	✓			✓	
[30]	Scalability			FLoRa, OMNeT++			✓	✓		
[31]	Scalability	Coarse-grained Scheduling			Light-weight scheduling	✓				
[32]	Scalability	Machine Learning		MATLAB	Variable Hysteresis	✓	✓			
[33]	Scalability				Integer Linear Programming	✓		✓	✓	
[34]	Scalability			NS-3						
[35]	Scalability	Mathematical Optimization		Monte Carlo	Stochastic Geometry	✓			✓	
[36]	Scalability		✓	✓					✓	
[37]	Throughput	Waterfalling Technique				✓	✓	✓	✓	
[38]	Throughput	Constrained Optimization			Gradient Projection Method	✓				
[39]	Throughput			LoRaSim			✓			
[40]	Throughput	Constrained Optimization			Markov-meta heuristics					
[41]	Throughput	Machine Learning			Reinforcement Learning	✓				
[42]	Throughput			MATLAB		✓		✓		
[44]	Throughput	Machine Learning		NS-3	Maximum Likelihood Estimation		✓		✓	✓
[45]	Throughput	Mathematical Optimization		MATLAB	Linear and Quadratic Approximation		✓			✓
[46]	Energy efficiency			FLoRa, OMNeT++	Integer Linear Programming					
[48]	Energy efficiency	Constrained Optimization		NS-3	Genetic Algorithm	✓			✓	✓
[49]	Energy efficiency			✓	✓	✓				
[50]	Energy efficiency	Mathematical Optimization		LoRaSim	Mixed Integer Linear Programming	✓		✓		✓
[51]	Energy efficiency	Constrained Optimization		MATLAB	Distributed Genetic Algorithm	✓		✓		

## Appendix A

**Table A1:** Acronyms used in this paper.

Acronym	Description
ACK	Acknowledge
ADR	Adaptive Data Rate
Bps	Bits per second
BW	Bandwidth
CF	Carrier Frequency
CR	Code Rate
CSS	Chirp Spread Spectrum
DL	Downlink
DER	Data Extraction Rate
FEC	Forward Error Correction
IoT	Internet of Things
JS	Join Server
LoRa	Long Range
LoRaWAN	Long Range Wide Area Network
LPWAN	Low Power Wide Area Network
MAC	Medium Access Control
NB-IoT	Narrow band Internet of Things
NS	Network Server
OTAA	Over-the-Air Activation
PDR	Packet Delivery Ratio
PER	Packet Error Rate
PLR	Packet Loss Ratio
RF	Radio Frequency
RW	Receive Window
SF	Spreading Factor
SNR	Signal to Noise Ratio
ToA	Time on Air
TDC	Transmission Duty Cycle
TP	Transmission Power
